# Energy Dissipation and Damage Evolution during Dynamic Fracture of Muddy Siltstones Containing Initial Damage under the Freeze Thaw Effect

**DOI:** 10.3390/ma16010120

**Published:** 2022-12-22

**Authors:** Yufei Jia, Yuxin Bai, Dong Xia, Fuping Li, Bing Liang

**Affiliations:** 1 College of Mining Engineering, North China University of Science and Technology, Tangshan 063210, China; 2 Hebei Province Mining Industry Development with Safe Technology Priority Laboratory, North China University of Science and Technology, Tangshan 063210, China; 3 Hebei Industrial Technology Institute of Mine Ecological Remediation, North China University of Science and Technology, Tangshan 063210, China

**Keywords:** freeze–thaw, SHPB, initial damage, energy distribution, fractal dimension, muddy siltstones

## Abstract

This research aims to evaluate the influences of the freeze–thaw (F-T) effect on the energy dissipation mechanism and damage evolution characteristics of muddy siltstones containing initial damage. At first, four initial damage levels were achieved by applying different impact loads to the intact rock, and the damage stresses for levels I, II, III, and IV initial damage were 9.80 Mpa, 17.00 Mpa, 23.34 Mpa, and 32.54 Mpa, respectively. Then dynamic compression tests were conducted on the muddy siltstones containing initial damage after 0, 5, 10, 15, 20, 25, 30, and 40 F-T cycles in the temperature range from −20 to 20 °C. The damage variable of the muddy siltstones was determined by studying energy distribution during fracture of the rock. The damage evolution characteristics of the muddy siltstone containing initial damage under the F-T effect were explored combined with the fractal theory. Test results show that (1) the dynamic compressive strength of the muddy siltstones decreases exponentially with the increasing number of F-T cycles; the dynamic compressive strength of muddy siltstone with different initial damage decreased by 54.9%, 48.4%, 39.4%, 42.5%, and 44.5% after 40 freeze-thaws, respectively, compared with that of intact. (2) The absorbed energy, reflected energy, and transmitted energy of the muddy siltstones subject to different levels of initial damage exhibit step-like changes under the effect of F-T cycles and the rate of decrease in absorbed energy decreases in the late stage of F-T cycles. (3) Both the damage variable and the fractal dimension of the muddy siltstones show an increasing trend with an increase in the number of F-T cycles, and it is more difficult for damage to become superimposed as the damage accumulates to that range causing fatigue (the damage variables ranged from 0.73 to 0.97) while the fractal dimension of the fracture surfaces in the rock still increases. (4) With the gradual increase in the damage variable, the energy absorption density of the rock is negatively correlated with the fractal dimension of the rock fragments.

## 1. Introduction

The freeze–thaw (F-T) weathering caused by seasonal alternation and the diurnal cycle threatens the safety and stability of rock engineering in cold regions [1,2,3]. The sustained action of F-T weathering is one of the important causes of secondary geological disasters [4,5,6]. Therefore, after systematic research into the deterioration effect of F-T weathering on rocks, many scholars [7,8,9] found that the F-T weathering effect significantly decreases parameters including the strength, elastic modulus, mass, and longitudinal wave velocity and also affects the failure mode of a rock mass [10,11,12]. Factors including the moisture content, initial state, and temperature difference of rocks also exert certain influences on the deterioration degree of rocks, which complicates the damage characteristics of rocks after being F-T weathered [13,14,15,16]. Therefore, the F-T effect has become one of the important factors that affect the construction and operation of rock engineering works in cold regions.

During engineering construction including mining, road slopes, and tunnel excavation, blasting and mechanical excavation are the main rock-breakage modes. Part of the energy produced by the explosion of explosives or mechanical disturbance is used to break and exfoliate rocks, thus reaching the goal of engineering excavation, while the other part is transferred to surrounding rocks as stress waves, which inevitably causes certain initial damage to surrounding rocks [17,18]. When undertaking rock engineering works in cold regions, there are also problems pertaining to the dynamic mechanical properties and the dynamic failure of the rocks under impact load or stress pulses [19,20,21]. Dynamic load plays an important role in the failure process of frozen and thawed rocks [22]. On this basis, many scholars studied the dynamic damage characteristics of rocks under the F-T effect and suggested that the strength, elastic modulus, and internal microstructures of the rocks are significantly deteriorated in certain ranges of F-T cycles and strain rate [23,24,25,26,27]. Microdefects dispersed in rocks constantly evolve from a disordered to an ordered distribution when rocks are under load and the F-T effect, thus forming macrocracks. Finally, these macrocracks coalesce to larger cracks along one direction, causing overall failure and collapse of the rock. In thermodynamic terms, the deformation process of rocks is an irreversible process of energy dissipation, so the deformation and damage process of rocks is in essence a complete process of energy dissipation and release. Under impact load, the failure characteristics are closely related to dissipated energy of damage [28,29,30,31], making it necessary to study the energy dissipation and damage evolution characteristics of rocks containing initial damage under the coupling of external load and the F-T effect.

The review and analysis of numerous research findings indicate that much research focuses on the damage and deterioration characteristics of rocks due to the F-T effect under static load. Existing research seldom considers dynamic load, particularly damage evolution characteristics, energy dissipation, and their interaction of rocks containing initial damage under the F-T effect after experiencing excavation disturbance, which remains to be further studied. In view of this, taking muddy siltstones as test specimens, a split Hopkinson pressure bar (SHPB) test system was used to produce initial damage in the specimens, thus forming specimens containing different levels of initial damage. After conducting 0, 5, 10, 15, 20, 25, 30, and 40 cyclic F-T tests on the intact rock specimens and saturated specimens containing different levels of initial damage, dynamic compression tests were conducted under identical conditions. The dynamic properties and energy dissipation of the rocks containing initial damage under different numbers of F-T cycles were investigated. In addition, the damage variable was determined from the perspective of energy. Finally, the mechanism relationship between energy dissipation and damage evolution in rocks with initial damage under the F-T effect is explained according to the evolution rule between rock damage variables, energy absorption density, and its fragmentation fractal characteristics.

## 2. Materials and Methods

### 2.1. Materials

**Figure 1:**
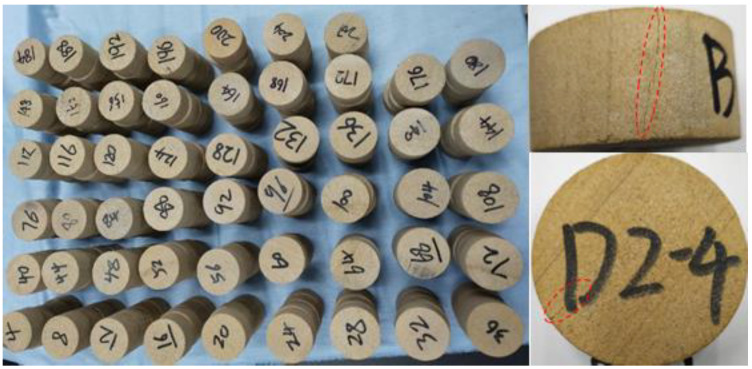
Rock specimens and the defective sample.

The muddy siltstones used in the experiment were taken from the north slope of the BaiLi Tan open-pit coal mine. The rock was machined into cylindrical rock specimens measuring φ 50 mm × 25 mm according to the method recommended by the International Society for Rock Mechanics (ISRM). The machined rock specimens were ground and leveled to ensure that the non-parallelism of the two end faces did not exceed 0.05 mm. The end faces should be vertical to the axis of the specimens, with a maximum deviation no greater than 0.25°. Rock specimens with visible defects and large differences in the mass and longitudinal wave velocity were excluded to reduce rock specimens’ heterogeneity. The rock specimens and the defective sample are shown in Figure 1. Basic physical parameters of the rock specimens including the uniaxial compressive strength and elastic modulus are listed in Table 1.

### 2.2. Test Device

**Figure 2:**
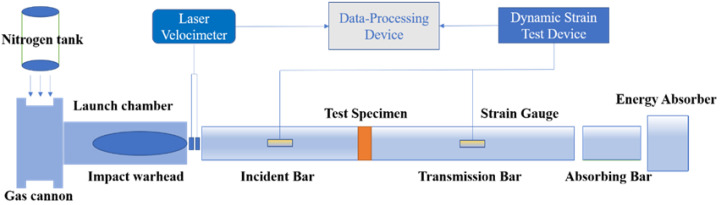
SHPB test device.

According to the method recommended by the ISRM, a SHPB test system was used to conduct dynamic compression tests on the rock specimens. The test system was composed of a liquid nitrogen tank, an impact warhead, an incident bar, a transmission bar, ultrahigh dynamic strain gauges, an oscilloscope, and a data acquisition system. The sketch map of the test device is displayed in Figure 2. The bars in the SHPB test device were forged with alloy steel with a diameter of 50 mm, density of 7579 kg/m^3^, and elastic modulus of 204 GPa. In the test process, the signals and data recorded by the strain gauges on the incident and transmission bars were collected by using the data acquisition system to provide the basic parameters for calculating the dynamic parameters and studying the energy change characteristics of the rock specimens below.

### 2.3. Test Schemes

**Figure 3:**
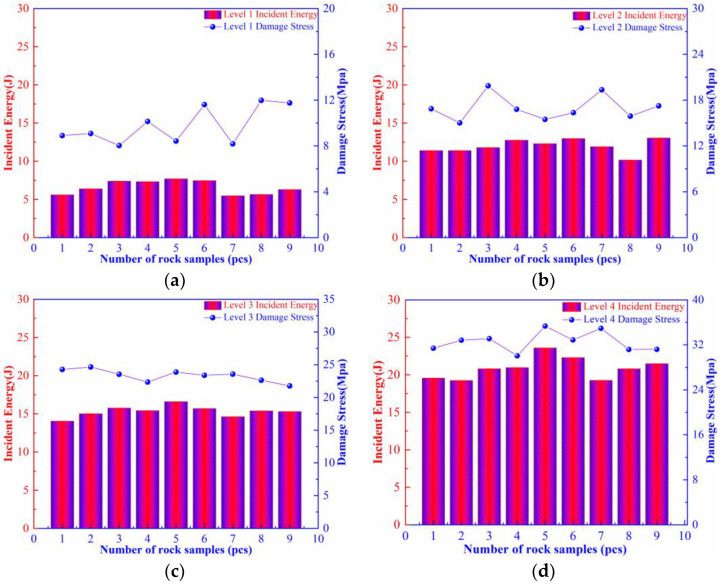
Peak stress and energy evolution statistics for rocks containing initial damage: (**a**) Level-I initial damage; (**b**) Level-II initial damage; (**c**) Level-III initial damage; (**d**) Level-IV initial damage.

**Figure 4:**
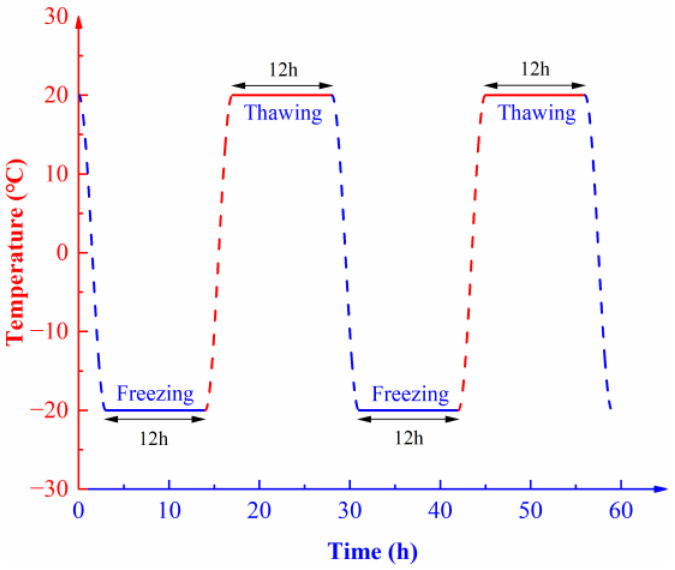
F-T scheme for the muddy siltstone specimen.

To simulate the generation process of initial damage in the rock due to disturbance including blasting vibration and mechanical excavation, the SHPB test system was used to generate initial damage within the intact rock specimens. Dynamic compression tests were conducted on the rock specimens containing different levels of initial damage after cyclic F-T tests. The specific test schemes are described as follows:

(1) Impact tests were performed on the rock specimens meeting the test requirements. The rock specimens were damaged when the incident-wave amplitude was 80 to 85 mV. The intact rock specimens (without initial damage) were impacted under 50%, 60%, 70%, and 80% of such an incident-wave amplitude to form the rock specimens containing different levels of initial damage, corresponding to levels I, II, III, and IV of initial damage. The changes in the incident energy and damage stress of the rock specimens containing initial damage are shown in Figure 3. At the four levels of initial damage, the average incident energy of the rock is 6.58 J, 11.96 J, 15.30 J, and 20.89 J and the average damage stress is 9.80 MPa, 17.00 MPa, 23.34 MPa, and 32.54 MPa, respectively.

(2) Intact rock specimens and saturated rock specimens containing different levels of initial damage were subjected to 0, 5, 10, 15, 20, 25, 30, and 40 F-T cycles under conditions of freezing and thawing both for 12 h within a F-T temperature range from −20 to 20 °C. The cyclic F-T process is shown in Figure 4.

(3) The rock specimens treated as described above were subjected to dynamic compression tests under the same conditions.

## 3. Test Results and Analysis

### 3.1. Dynamic Properties

**Figure 5:**
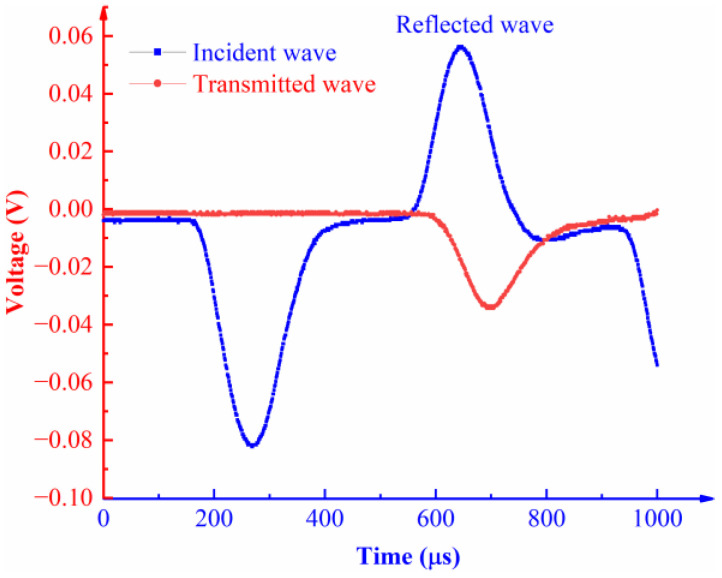
Typical voltage-time waveform.

Keeping the impact load constant is the premise of ensuring reliable data from dynamic compression tests. According to the test results, typical waveforms of the rock specimens are obtained, as illustrated in Figure 5.

Based on two basic assumptions of the SHPB impact load tests and the one-dimensional stress wave theory, the stress σ(t), strain ε(t), and strain rate $\dot{\varepsilon }(t)$ of the rock specimens under impact load were calculated using the three-wave method and Equations (1)–(3) [32]:

(1) $$\sigma (t)=\frac{E_{0}A_{0}}{2A_{s}}\left[\varepsilon_{I}(t)-\varepsilon_{R}(t)-\varepsilon_{T}(t)\right]$$

(2) $$\varepsilon (t)=\frac{C_{0}}{l_{s}}\int_{0}^{\tau }\left[\varepsilon_{I}(t)-\varepsilon_{R}(t)-\varepsilon_{T}(t)\right]dt$$

(3) $$\dot{\varepsilon }(t)=\frac{C_{0}}{l_{s}}\left[\varepsilon_{I}(t)-\varepsilon_{R}(t)-\varepsilon_{T}(t)\right]$$

where $E_{0}$, $C_{0}$, and $A_{0}$ separately represent the elastic modulus (GPa; m/s; mm^2^), propagation velocity of elastic waves in bars (m/s), and cross-sectional area of bars (mm^2^); $A_{S}$ and $l_{S}$ denote the cross-sectional area (mm^2^) and height (mm) of the rock specimens; $\varepsilon_{I}(t)$, $\varepsilon_{R}(t)$, and $\varepsilon_{T}(t)$ refer to the incident strain, reflected strain, and transmitted strain, respectively; t is the duration of elastic waves (s). Therein, $C_{0}$ is calculated using Equation (4):

(4) $$C_{0}=\sqrt{E_{0}/\rho_{0}}$$

where $\rho_{0}$ denotes the density (kg/m^3^) of the elastic bar.

The stress σ(t), strain ε(t), and strain rate $\dot{\varepsilon }(t)$ of the muddy siltstones after the different numbers of F-T cycles were obtained using Equations (1)–(3), as listed in Table 2.

Table 2 shows that the dynamic compressive strength of the rock gradually reduces while the peak strain and average strain rate gradually increase with the increasing number of F-T cycles. After 40 F-T cycles, the dynamic compressive strength of the rock specimens decreases from 49.48 MPa in the drying state to 22.33 MPa, which is 45.13% of the initial value. The peak strain and average strain rate of the rock specimens separately increased by 51.88% and 44.72% separately from 8.52‰ and 69.87 (1/s) in the drying state to 12.94‰ and 101.12 (1/s). The mechanical parameters including dynamic compressive strength and tensile strength of the rock are positively correlated with the strain rate [33,34,35] while the dynamic compressive strength in the test results is negatively correlated with the average strain rate. The reason for this phenomenon is that the sustained freeze–thaw effect changes the structural stiffness of the rock sample itself, causing a decrease in the ability of the sample to resist deformation, and thus the peak strain and strain rate of the rock sample gradually increase after freeze–thaw under constant impact gas pressure.

**Figure 6:**
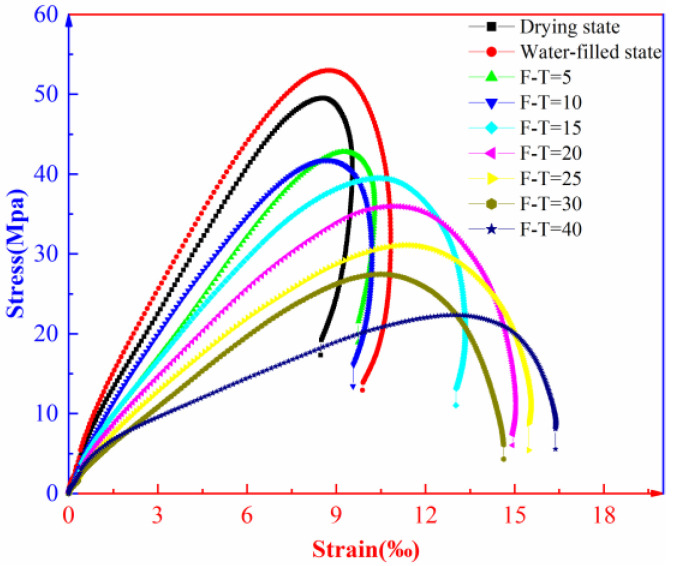
Dynamic stress–strain curves of muddy siltstones after F-T cycles.

The dynamic stress–strain curves of the intact muddy siltstones under the different numbers of F-T cycles are illustrated in Figure 6.

As shown in Figure 6, the F-T effect greatly influences the deformation parameters including the peak strain, elastic modulus, and total strain of the rock under impact load. The failure process of the rock under impact load can be divided into an initial compaction stage, an elastic deformation stage, a yield stage, and a fracturing stage. In the elastic deformation stage, the elastic strain of the rock gradually increases while the elastic modulus decreases with the increasing number of F-T cycles. In the yield stage, the plastic strain increases with the increasing number of F-T cycles. This is because the F-T effect damages some bonded structures in the rock and such damage becomes more significant with the increasing number of F-T cycles. In the fracturing stage, strain rebound occurs to the post-peak deformation of the rock after fewer than 10 F-T cycles; when the number of F-T cycles exceeds 30, the post-peak deformation shows significant strain softening. Post-peak deformation of the rock exhibits a transition from elastic rebound to strain softening. This is because the F-T effect changes the internal structures of the rock, and the effect becomes more obvious with the increasing number of F-T cycles.

**Figure 7:**
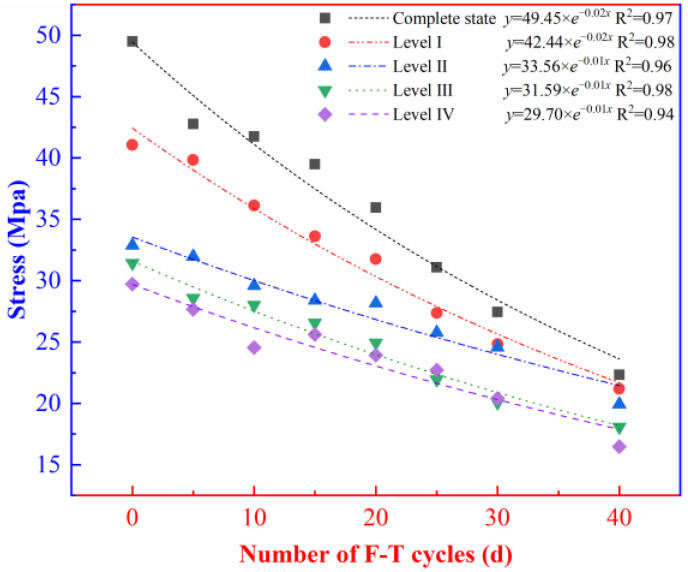
Scatter plot of the number of F-T cycles and peak stress.

To evaluate the influences of initial damage on the dynamic properties of the rock under the F-T effect, the scatter plot of the number of F-T cycles with the peak strength of the rock containing initial damage was plotted (Figure 7).

As illustrated in Figure 7, the presence of initial damage greatly affects the peak strength of the rock. The peak strength shows a decrease trend with the increasing level of initial damage. The strength of the rock containing initial damage decreases exponentially with the number of F-T cycles under the F-T effect. After 40 F-T cycles, the peak strength of the intact rock and the rock containing level-I, II, III, and IV initial damage decreases by 54.9%, 48.4%, 39.4%, 42.5%, and 44.5%, respectively, compared with that before application of any F-T cycles. Before the F-T cycles, the peak strength of the rock containing level-I, II, III, and IV initial damage decreases separately by 17.0%, 33.6%, 36.5%, and 39.9% compared with that of intact rock. As the level of initial damage is increased, the decay degree of peak stresses in the rocks under the F-T effect decreases, indicating that the presence of initial damage may influence the deterioration rate of the rock under the F-T effect.

### 3.2. Energy Dissipation

In the SHPB test, the gas cannon was used to strike the incident bar at a constant rate to produce the incident energy (*W_I_*), reflected energy (*W_R_*), and transmitted energy (*W_T_*). Then, the absorbed energy (*W_S_*) of the rock specimens in the impact process is calculated using Equations (5) and (6). The incident energy (*W_I_*), reflected energy (*W_R_*), transmitted energy (*W_T_*), absorbed energy (*W_S_*), absorbed energy per unit volume (*E_V_*), and transmission efficiency of reflected energy (*E_R_*) can be calculated using Equations (5)–(8):

(5) $$W_{i}=\frac{A_{0}C_{0}}{E_{0}}\int \sigma_{i}^{2}(t)dt\;i=I,R,T$$

(6) $$W_{S}=W_{I}-W_{R}-W_{T}$$

(7) $$E_{V}=\frac{W_{S}}{V_{S}}$$

(8) $$E_{R}=\frac{W_{R}}{W_{I}}$$

where $\sigma_{I}\left(t\right)$, $\sigma_{R}\left(t\right)$, and $\sigma_{T}\left(t\right)$ denote the incident waves, reflected waves, and transmitted waves, respectively; *E_V_* represents the volume (mm^3^) of the specimens.

To estimate the influences of the F-T effect on the energy distribution of the rock specimens containing initial damage, the incident energy is kept quasi-constant within the range from 33 J to 37 J in the test process.

**Figure 8:**
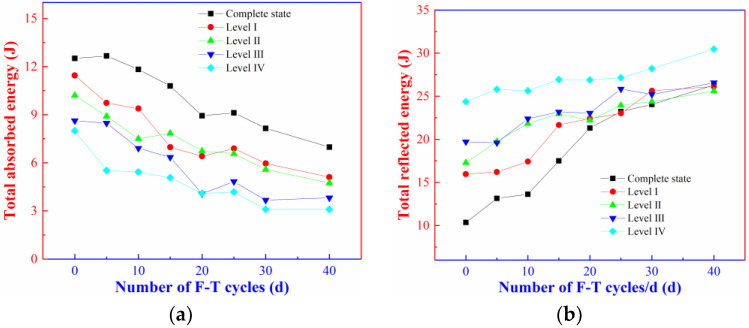

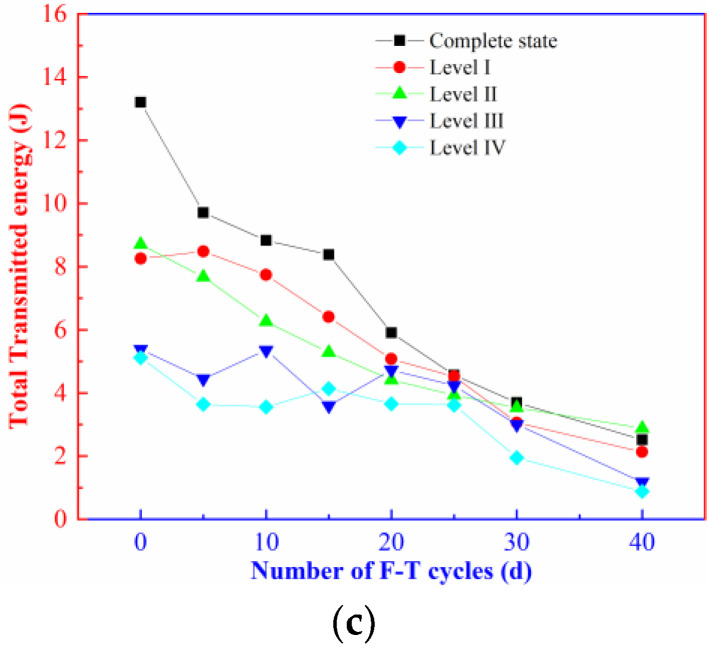
Distribution curves of the number of F-T cycles with energy: (**a**) Total absorbed energy; (**b**) Total reflected energy; (**c**) Total transmitted energy.

Changes in the reflected energy (*W_R_*), transmitted energy (*W_T_*), and absorbed energy (*W_S_*) of the rock specimens containing initial damage after the different numbers of the F-T cycles are illustrated in Figure 8.

Figure 8 show that the F-T effect affects the absorbed energy, reflected energy, and transmitted energy of the intact rock and rock specimens containing different levels of initial damage under the impact load to different extents. Under impact load, the absorbed energy is mainly dissipated in the propagation and extension of microcracks and generation of new cracks and fractures (not considering the kinetic energy, thermal energy, or energy in other forms) in the rock. With the increasing number of F-T cycles, the energy absorbed in the rock specimens shows a decreasing trend, and the absorbed energy of intact rock and rock specimens containing level-I, II, III, and IV initial damage separately decrease by 44.21%, 55.37%, 53.53%, 55.74%, and 61.20% compared with that before the F-T cycles. The phenomena have also been observed by Ma [31] and Meng [36]. Figure 8a demonstrates that the absorbed energy in each group of rock specimens decreases significantly in the first 20 F-T cycles and continues to decrease albeit with a gradually decreased amplitude with the increasing number of F-T cycles. After a same number of F-T cycles, the absorbed energy of the rock specimens shows a decreasing trend with the increasing level of initial damage and decay amplitude gradually decreases. Such phenomena indicate that the ability of the rock samples containing initial damage to resist rupture gradually decreases under the F-T effect, and the magnitude of this decrease is clearly related to the extent of damage accumulation in the samples themselves.

By observing Figure 8b,c, it is found that the F-T effect also significantly influences the reflected energy and transmitted energy of the rock. With the increasing number of F-T cycles, the reflected energy increases while the transmitted energy decreases, and they change to different extents in the rock specimens containing different levels of initial damage. After 40 F-T cycles, the reflected energy of the intact rock and the rock specimens containing level-III initial damage separately increases by 153.80% and 34.79% and the transmitted energy separately decreases by 80.90% and 78.08%. The differences between the steps of the reflected energy and transmitted energy of the rock specimens containing different levels of initial damage decrease. The presence of initial damage accelerates the deterioration of the F-T effect on the rock, which is equivalent to reducing the number of F-T cycles to form the same degree of damage in the rock sample.

### 3.3. Damage Evolution Characteristics

According to the theory of stress wave propagation, the stress waves produce reflected waves and transmitted waves because of the difference in impedance at the interface between two materials [37]. Therefore, the damage variable can be defined based on the wave impedance of materials. According to the one-dimensional stress wave theory [38], Equation (9) holds such that:

(9) $$\sigma_{R}(t)=\lambda \sigma_{I}(t)$$

where $\lambda =\left(\rho_{1}C_{1}-\rho_{2}C_{2}\right)/\left(\rho_{2}C_{2}+\rho_{1}C_{1}\right)$ represents the reflection coefficient of the stress waves entering from the elastic bar to the rock; $\rho_{1}C_{1}$ and $\rho_{2}C_{2}$ separately denote wave impedances (MPa/s) of the elastic bar and the rock; ρ represents the density (kg/m^3^); C is the longitudinal wave velocity (m/s).

Equation (10) can be deduced by combining Equations (5), (8) and (9):

(10) $$E_{R}=\frac{W_{R}}{W_{I}}=\frac{\sigma_{R}^{3}(t)\cdot {\sigma^{\prime }}_{I}(t)}{\sigma_{I}^{3}(t)\cdot {\sigma^{\prime }}_{R}(t)}=\lambda^{3}\cdot \frac{1}{\lambda }=\lambda^{2}$$

Equation (11) giving the wave impedance of the rock can be deduced according to the reflection coefficient and Equation (10).

(11) $$\rho_{2}C_{2}=\frac{1-\lambda }{1+\lambda }\rho_{1}C_{1}$$

The wave impedance ${\left(\rho_{2}C_{2}\right)}_{nm}$ of the rock containing initial damage after the different numbers of the F-T cycles can be calculated according to the transmission efficiency of reflected energy (*E_R_*) (Equation (12)).

(12) $${\left(\rho_{2}C_{2}\right)}_{nm}=\frac{1-\sqrt[{}]{E_{Rnm}}}{1+\sqrt[{}]{E_{Rnm}}}\rho_{1}C_{1}$$

The damage variable (*D_nm_*) of the rock is determined using Equation (13).

(13) $$D_{nm}=1-{\left(\frac{\bar{\rho C}}{\rho C}\right)}^{\alpha }=1-{\left(\frac{\left(1-\sqrt[{}]{E_{Rnm}})(1+\sqrt[{}]{E_{R}}\right)}{\left(1+\sqrt[{}]{E_{Rnm}})(1-\sqrt[{}]{E_{R}}\right)}\right)}^{\alpha }$$

where $\bar{\rho C}$ and ρC denote the wave impedances of the rock specimens at a damage state and the initial state, respectively; n and m separately represent the number of F-T cycles and the level of initial damage; α is a coefficient to be determined (it is set to 1.6 according to Jin [39]).

According to Equation (13) and the test results, the damage variable (*D_nm_*) of the rock specimens containing initial damage due to the F-T effect under impact load is calculated.

**Figure 9:**
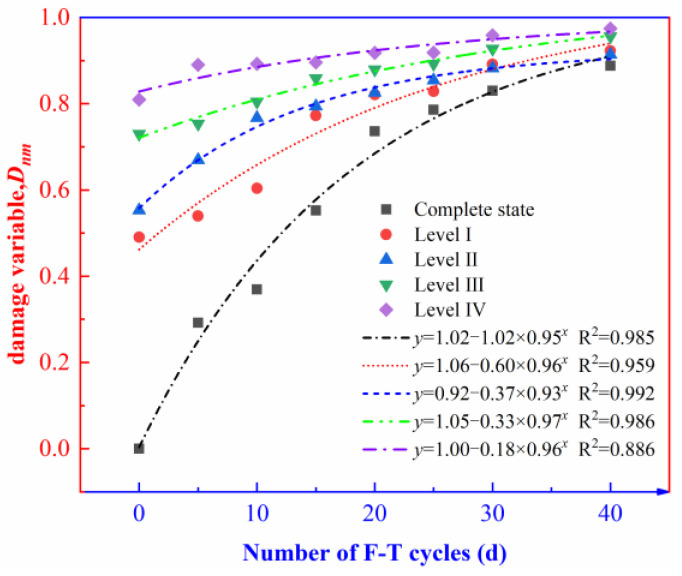
A plot of the number of F-T cycles in function of damage variables due to the F-T effect.

The relationships between the number of F-T cycles and the damage variable (*D_nm_*) of the rock containing initial damage due to the F-T effect were plotted according to the test results (Figure 9).

Figure 9 implies that the F-T effect significantly influences the damage variables of both the intact rock and the rock specimens containing level-I, II, III, and IV initial damage. The damage variable of the rock specimens shows an exponentially increasing trend with the increasing number of F-T cycles. The same phenomenon has also been noticed by Wang [40], Liu [41], and Niu [42]. The presence of initial damage affects the damage evolution of the rock specimens under the F-T effect and the slope of the variable damage curve diminishes as the initial damage level increases. The relationship between the number of F-T cycles and the damage variable (*D_nm_*) of the rock specimens can be fitted using an exponential model:

(14) $$y=a-b\times c^{x}$$

Fitting parameters a, b, c, and $R^{2}$ are listed in Table 3.

Coefficients a and b in the fitting equation determine the value of the damage variable (*D_nm_*) of the rock specimens before being frozen and thawed, and c determines the rate of change of the damage variable (*D_nm_*). In Table 3 a and c change slightly while b decreases with the increasing level of initial damage. By taking the first-order derivative of Equation (14), the following can be obtained:

(15) y(0)=a−b

(16) $$y^{\prime }=-b\cdot c^{x}\cdot \ln\left(c\right)$$

If the number of F-T cycles of the intact rock is $x_{0}$, the damage variable $y_{0}$ is

(17) $$y_{0}=a_{0}-b_{0}\times c_{0}{}^{x_{0}}$$

If $y_{0}$ is fixed, the number $x_{m}$ of F-T cycles to allow the rock specimens containing different levels of initial damage to reach $y_{0}$ is

(18) $$x_{m}=\log_{c_{m}}\frac{a_{m}-y_{0}}{b_{m}}$$

By using Equation (18), the first-order derivative ${y^{\prime }}_{m}$ of different levels of initial damage after $x_{m}$ F-T cycles is calculated.

(19) $${y^{\prime }}_{m}=(y_{0}-a_{m})\times \ln(c_{m})$$

It can be seen from Equation (19) that the first-order derivative for the fitting curve of the damage variable of the rock does not have a direct relationship with *b*, the value of which gradually decreases with the increasing level of initial damage. Therefore, the rate of change of the damage variable with the number of F-T cycles also fails to have a direct correlation with the level of initial damage. The damages variable of the rock specimens containing level-II, III, and IV initial damage (0.553, 0.730, and 0.809) are similar to those of the intact rock after 15, 20, and 30 F-T cycles (0.553, 0.736, and 0.830). The damage variable of the rock specimens in Figure 9 grows exponentially with the gradual increase in the number of F-T cycles. Therefore, the influences of initial damage on the damage evolution of the rock under the F-T effect are manifest in two ways: (1) the initial damage increases the damage accumulation in the rock specimens in that period without an F-T effect and shortens the time taken by the rock to reach the same extent of damage under the F-T effect, thus indirectly facilitating the efficiency of the F-T cycles on rock deterioration; (2) With the initial damage level increasing, the damage inside the rock sample accumulates more and more, and its own skeleton structure is heavily deteriorated. It is difficult to further superimpose freeze-thaw damage, and this rule is also conformed to the growth trend of the late exponential asymptotic model.

**Figure 10:**
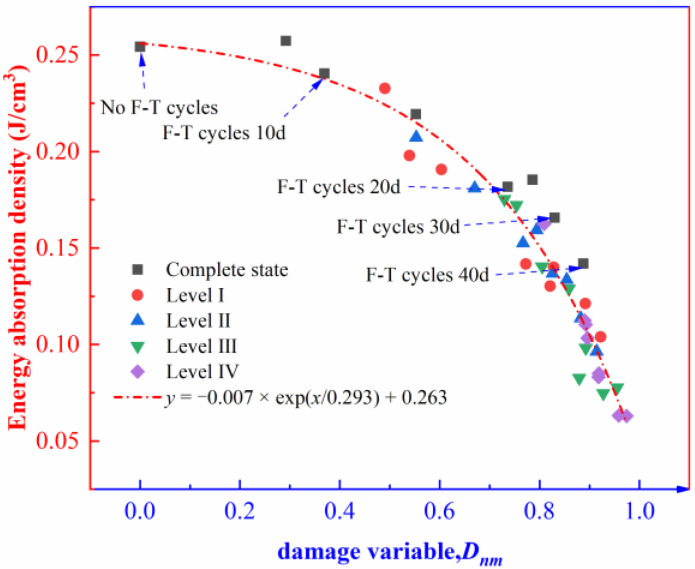
The distribution of the energy absorption density and the damage variable.

To study further the damage evolution characteristics of the rock specimens containing initial damage, the distribution of the energy absorption density and the damage variable is plotted (Figure 10).

As shown in Figure 10, the energy absorption density of the rock decreases exponentially with the increase in the damage variable. When the damage variable is less than 0.73, the energy absorption density is reduced by 28% from 0.25 J/cm^3^ to 0.18 J/cm^3^; when the damage variable is between 0.73 and 0.97, the energy absorption density is reduced by 67% from 0.18 J/cm^3^ to 0.06 J/cm^3^. The energy absorption density of the rock specimens containing different levels of initial damage is mainly concentrated in the range corresponding to the damage variable from 0.73 to 0.97, during which the decrease in energy absorption density accelerates. Based on the above analysis, the damage variables of the rock specimens containing different levels of initial damage tend to stabilize under the F-T effect. After 40 F-T cycles, the energy needed for the failure of the rock specimens decreases successively with the rising level of initial damage. The result indicates that after damage accumulates to a certain extent, the internal structure of the rock is deteriorated, thus causing low-energy fatigue albeit leading to significant damage at failure of the rock.

### 3.4. Fractal Features of Rock Fragments

**Figure 11:**
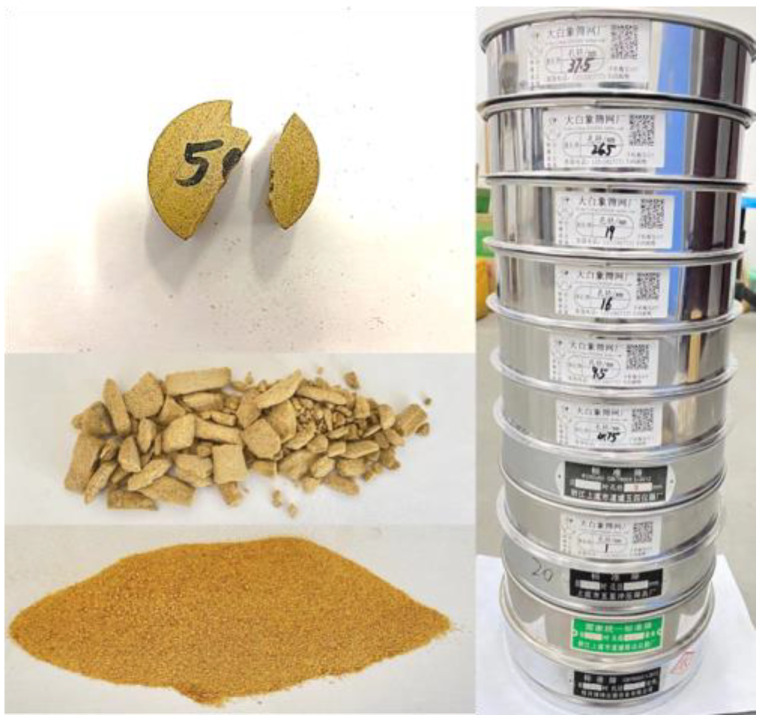
Grain size sieving and standard sieves.

Under the external load, the energy in the rocks is rapidly accumulated, transformed, and released, thus inducing the propagation, extension, gathering, and coalescence of internal microcracks and micropores and fragmentation of the rocks. The evolution of the internal spatial structures of the rocks shows the self-similarity, which inevitably causes broken rock fragments to exhibit a corresponding self-similarity. Under impact load, the fractal dimension (*D_s_*) of the rock fragments is related to the internal structural change of the rocks, so the fractal dimension (*D_s_*) can be used to quantify the changes in complex structures in the rock. Therefore, the fractal theory and the law governing the dissipation of energy were introduced to perform in-depth research on the structural deterioration process of the rock specimens containing initial damage under the F-T effect. Standard sieves with mesh apertures between 0.154 and 37.5 mm were used to screen the broken rock fragments, and mass of rock fragments screened by each sieve was measured. The standard sieves and some screened rock specimens are shown in Figure 11.

The fragment distribution equation of the rock under the impact load is [43]

(20) $$M(x)/M_{T}={\left(x/x_{m}\right)}^{3-D_{s}}$$

(21) $$D_{S}=3-A$$

where M(x) and $M_{T}$ separately represent the cumulative mass and total mass (g) of the rock fragments; x and $x_{m}$ separately denote the particle size and maximum particle size (mm) of the rock fragments; $D_{S}$ is the fractal dimension of the rocks.

**Figure 12:**
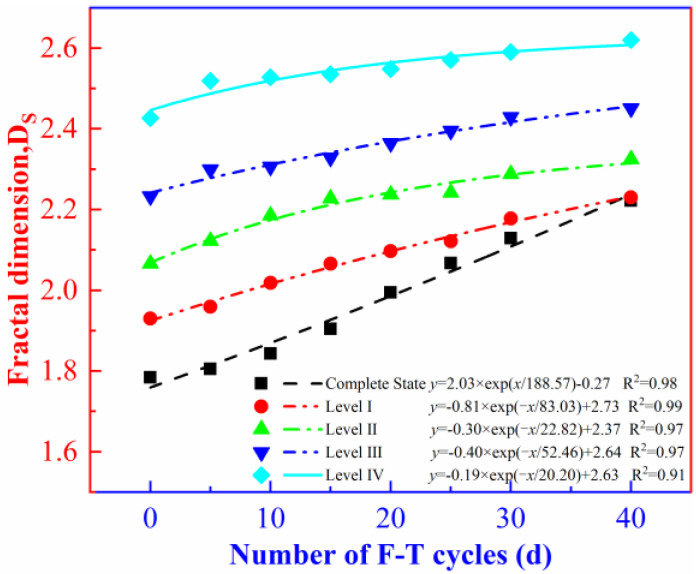
Fractal dimension curves of rocks with different levels of initial damage under the F-T effect.

The lg($M(x)/M_{T}$)−lg$\left(x/x_{m}\right)$ double-logarithm coordinate system is constructed by taking logarithms in both sides of Equation (20). Then, the slope is calculated through the linear fitting. In this way, the fractal dimension (*D_s_*) of the rock fragments can be calculated using Equation (21). The calculated fractal dimensions of the rock specimens containing different levels of initial damage under the F-T effect was plotted. (Figure 12).

It can be seen from Figure 12 that the fractal dimension (*D_s_*) of the rock fragments increases with the increasing number of F-T cycles. Similar rules have also been obtained by Song [44] and Li [45]. The intact rock, before being frozen and thawed, is found to have the minimum fractal dimension (*D_s_*), which gradually increases with the increasing level of initial damage. After 40 F-T cycles, the fractal dimension (*D_s_*) of each group of the rock specimens always shows an increasing trend. With the increasing level of initial damage, the slope of the fractal dimension (*D_s_*) fitting curve of the rock fragment gradually decreases, while the higher the level of initial damage, the greater the corresponding fractal dimension (*D_s_*). Under the action of a same number of F-T cycles, the average particle size of the rock fragments under the impact load decreases with the increasing level of initial damage.

As shown in Figure 9 and Figure 12, the damage variable of the rock and the fractal dimension of the rock fragments both exponentially increase with the increasing number of F-T cycles and the growth rates of the two decreases with the increasing level of initial damage. This indicates that the deterioration of the internal spatial structures and the fragmentation degree of the rocks show a certain self-similarity.

**Figure 13:**
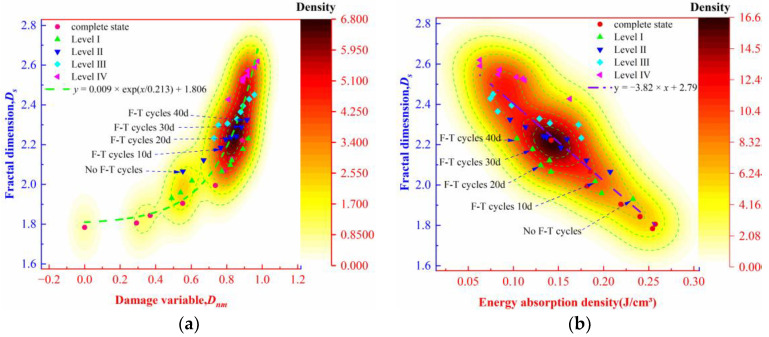
Density distribution of fractal dimension fragments: (a) Damage variable (D); (b) Energy absorption density.

The damage and fracture of the rocks are a process of energy accumulation and dissipation. On this basis, the density distributions of the fractal dimension with the damage variable and energy absorption density of the rock specimens were plotted (Figure 13a,b).

It can be seen from Figure 13a,b that the fractal dimension of the rock fragments increases with the increase in the damage variable and the decrease in the energy absorption density. According to Figure 13a, the density contour above 3.400 shows the rapid fractal dimension, where the fractal dimension rapidly increases from 2.23 to 2.62. In Figure 13b, the fractal dimension of the rock fragments is mainly distributed above the 8.325 density contour, where the fractal dimension also increases from 2.23 to 2.62. Therefore, the F-T effect induces a greater deterioration efficiency and a more significant fragmentation effect on the rock containing initial damage compared with the intact rock. After the damage accumulates to values concomitant with fatigue, that is, with a damage variable between 0.73 and 0.97, the damage accumulation efficiency reduces significantly in the rock and the energy absorption density greatly decreases while the fractal dimension increases significantly. Many studies have found that the fractal dimension of the rock fragments is directly proportional to the energy dissipation density [46,47]; however, the two show an inverse relationship in the research because of the persistent damage accumulation in the rock. The phenomenon is further explained based on the law of conservation of energy. At first, the rock containing initial damage absorbs energy in the F-T process to facilitate crack development, which at the same time decreases the strain energy needed at fracture, so that the greater the accumulation of damage, the lower the energy needed for rock fracturing. Then, the persistent increase in the fractal dimension of the rock containing initial damage under the F-T effect is a result of the energy release after the failure of the rock. That is, the fractal dimension of the rock fragments is the result of the joint action of the damage accumulation and the fracture, justifying the study of the fractal dimension of the rocks during the evolution of damage thereto.

## 4. Conclusions

To study the influences of the F-T effect on the dynamic properties, energy dissipation, and damage evolution characteristics of the muddy siltstones containing initial damage, 0, 5, 10, 15, 20, 25, 30, and 40 F-T cycles were conducted on intact rock specimens and saturated specimens containing different levels of initial damage. The tests were conducted under conditions of freezing and thawing separately for 12 h within the temperature range from −20 to 20 °C. Thereafter, these specimens were subjected to impact load tests under the same conditions. According to the test results, the following conclusions were drawn:

(1) The dynamic compressive strength of the muddy siltstones under the F-T effect shows a decreasing trend with the increasing level of initial damage. After 40 F-T cycles, the peak strength of the intact rock and rock specimens containing level-I, II, III, and IV initial damage decreases by 54.9%, 48.4%, 39.4%, 42.5%, and 44.5%, respectively, compared with that before freezing and thawing. The presence of initial damage exerts significant influences on the strength and rate of deterioration of the rock under the F-T effect.
(2) The absorbed energy and transmitted energy of the muddy siltstones reduce, while the reflected energy gradually increases, with the increasing number of F-T cycles. The presence of initial damage causes obvious step-like changes of the energy distribution. After 20 F-T cycles, the decrease in the absorbed energy in the rock gradually slows and less energy is needed for fracture. At the same time, the difference between the steps of the transmitted energy and reflected energy gradually narrows. After a given number of F-T cycles, the time to the decrease in amplitude of absorbed energy in the rock is advanced with the increasing level of initial damage. The presence of initial damage facilitates the deterioration efficiency of the rock due to the F-T effect and reduces the number of F-T cycles that cause the same amount of damage.
(3) The damage variable of the muddy siltstones shows an exponential increase trend with the increasing number of F-T cycles. Under the F-T effect, the rate of growth of the damage variable of the rock diminishes with the increasing level of initial damage. The initial damage does not directly promote the evolution of damage in the muddy siltstones under the F-T effect but does increase the extent of the damage accumulation of the rock in that time with no F-T cycling, thus indirectly accelerating the F-T deterioration efficiency. With the gradual increase in the level of initial damage, the skeleton structure of the rock is deteriorated, and it is more difficult for the F-T damage to be further superimposed thereon.
(4) Under the F-T effect, the energy absorption density of the muddy siltstones containing initial damage gradually decreases with the increasing damage variable. The energy absorption density reduces by 67% when the damage variable is between 0.73 and 0.97. When the damage accumulation in the muddy siltstones exceeds a certain amount, the skeleton structure of the rock is deteriorated and the rock undergoes low-energy fatigue, eventually leading to severe damage at failure.
(5) The fractal dimension of the muddy siltstones containing different levels of initial damage gradually increases with the increasing number of F-T cycles and damage variable, while it exhibits an inverse relationship with the energy absorption density. The fractal dimension of the muddy siltstones increases significantly when the damage variable is within the range from 0.73 to 0.97.

## Figures and Tables

**Table 1:** Basic physical parameters of siltstone.

Average Longitudinal Wave Velocity (m/s)	Saturation Density (g/cm^3^)	Drying Density (g/cm^3^)	Porosity (%)	Elastic Modulus (GPa)	Uniaxial Compressive Strength (MPa)
1886	2.31	2.20	11.6	6.03	45.76

**Table 3:** Exponential fitting results.

Fitting Parameters	a	b	c	$R^{2}$
Complete state	1.02	1.02	0.95	0.985
I	1.06	0.60	0.96	0.959
II	0.92	0.37	0.93	0.992
III	1.05	0.33	0.97	0.986
IV	1.00	0.18	0.96	0.886

**Table 2:** Dynamic parameters of siltstones under the F-T effect.

Number of F-T Cycles *n*	Peak Strain (‰)	Peak Stress (MPa)	Average Strain Rate (1/s)
Drying state	8.52	49.48	69.87
Water-filled state	8.74	52.96	70.01
5	9.26	42.76	72.98
10	8.70	41.74	76.73
15	10.48	39.49	88.85
20	10.89	35.95	93.95
25	11.38	31.09	96.49
30	10.49	27.44	73.73
40	12.94	22.33	101.12

## Data Availability

The authors confirm that the data supporting the findings of this study are available within the article.
